# Reasons for unsafe abortion in Iran after pronatalist policy changes: a qualitative study

**DOI:** 10.1186/s12978-024-01929-4

**Published:** 2024-12-16

**Authors:** Arezoo Haseli, Nasrin Rahnejat, Dara Rasoal

**Affiliations:** 1 Family Health and Population Growth Research Center, Health Institute, Kermanshah University of Medical Sciences, Kermanshah, Iran; 2 Clinical Research Development Center, Motazedi Hospital, Kermanshah University of Medical Sciences, Kermanshah, Iran; 3 School of Health and Welfare, Dalarna University, Högskolegatan 2, 79188 Falun, Sweden

**Keywords:** Reproductive health, Stigma, Women’s experiences, Illegal abortion

## Abstract

**Background:**

In Iran, restrictive abortion laws have led to widespread unsafe abortions, posing significant health risks. The 2021 Family and Youth Protection Law further restricted access to reproductive health services in an effort to boost birth rates. The purpose of this qualitative study is to explore the reasons women sought abortions in an illegal context, based on their own experiences.

**Methods:**

This exploratory qualitative study involved in-depth interviews with 46 women in Kermanshah, Iran, between April and August 2024. All participants had experienced incomplete abortions after undergoing unsafe procedures. Data were analyzed using conventional content analysis and thematic analysis with MAXQDA 10 software to identify key themes in the women's experiences. To ensure the study's rigor, we applied Guba and Lincoln’s criteria, including credibility, dependability, confirmability, and transferability.

**Results:**

Five main themes emerged from the interviews: economic hardship, pursuit of a prosperous life, unstable marital relationships, health and fertility issues, and cultural factors. Economic challenges, such as unemployment and lack of basic necessities, were the most frequently cited reasons for seeking unsafe abortions. Health issues, including unplanned pregnancies and fear of fetal anomalies, also played a significant role, alongside cultural stigmas related to age, illegitimacy, and gender preferences.

**Conclusion:**

This study sheds light on the multifaceted factors driving unsafe abortions in Iran following pronatalist policy changes. A holistic approach is recommended to address the interconnected economic, social, and cultural challenges that contribute to this issue. By implementing such comprehensive strategies, policymakers and stakeholders can work to reduce the prevalence of unsafe abortion practices and foster improved health and well-being for women.

## Introduction

From the perspective of demography, fertility is known as the most important phenomenon that determines population fluctuations (sex and age structure of the population) [1]. The decline in population growth in Iran is influenced by a combination of economic, social, and cultural factors, with unsafe abortions being reported as one of the most significant contributors to this trend [2]. Abortion is defined as the termination of pregnancy, whether spontaneous, before the 22nd week of pregnancy, or induced [3]. The World Health Organization defines unsafe abortion as any procedure aimed at terminating a pregnancy that is performed by people lacking appropriate skills and/or in an unsanitary and non-medical environment [4]. Less than half of the 55.7 million abortions that occur annually in the world either by an unsafe provider, by an unsafe method, or both (6), and 97% of these occur in developing countries [5]. It is estimated that unsafe abortion is responsible for 7.9% of maternal deaths. They also lead to a large number of short-term and long-term complications, including bleeding and sepsis [6]. Today, abortion in Iran faces complex social issues and health care challenges. With abortion illegal, many women seek clandestine and unsafe abortions [7]. There are no official statistics on abortion in Iran due to the sensitive nature of this phenomenon and strict abortion laws. It is estimated that 300,000 to 600,000 illegal abortions are performed annually in Iran [8].

Abortion at the request of women is the most common reason for illegal abortions, typically carried out in secrecy using various methods in Iran. These include catheterization to open the cervix (involving the injection of alcohol, saline solution, or other substances into the uterus [9]; inserting foreign objects such as pieces of wood, clothes hangers, safety pins, or knitting hooks into the uterus; and injecting air with a syringe [10]. Sharp objects may also be inserted into the uterus, causing perforation of the uterus and amniotic sac, often resulting in watery discharge, which indicates the termination of pregnancy [11]. Other methods involve performing curettage or suction curettage in illegal centers, often under substandard and unsanitary conditions [9]. Harmful substances, both edible and injectable are sometimes used, including oral or vaginal prostaglandin compounds, metal salts, phosphorus, lead, petroleum products, cleaning solutions, and various herbal compounds [9, 12]. In some cases, the uterus is washed with toxins, or substances such as soap compounds and chemicals are inserted into the cervix [9]. Physical trauma is another means of inducing abortion, including self-inflicted blows to the abdomen, intense abdominal massages, jumping from heights, and lifting heavy objects [9].

With the approval of the Family and Youth Protection Law in Iran in 2021, strict regulations were introduced with the aim of increasing the population [13]. These measures included eliminating routine fetal health screenings, imposing strict security laws against abortion providers, restricting access to family planning services, banning permanent sterilization of both men and women, and prohibiting the free distribution of contraceptives, the implantation of contraceptive devices, and even the promotion of their use [14].

In Iran, sociocultural norms surrounding family composition and childbearing play a significant role in shaping couples' decisions about having children. These decisions are influenced by mutual relationships and an evaluation of factors such as economic stability, educational opportunities, and access to healthcare [15]. However, in recent years, these underlying conditions have worsened, creating challenges for families attempting to meet traditional expectations. Rising divorce rates and economic hardships, including high inflation and unemployment, have reduced the ability of many families to align with societal norms regarding childbearing[15]. Moreover, diminished hope for the future among young couples has further discouraged childbearing, pushing many to reconsider these norms [16, 17].

Therefore, the new population policies have raised significant concerns among reproductive health professionals, one of these concerns is the possibility of an increase in the rate of unsafe abortion and its related consequences due to the restriction of access to family planning and reproductive health services [18]. However, there is limited evidence on the impact of the Family and Youth Protection Law on the abortion situation in Iran. Therefore, the purpose of this qualitative study is to explore the reasons women sought abortions in an illegal context, based on their own experiences.

## Methods

This qualitative exploratory study utilized data collected through in-depth, open-ended interviews with 46 cisgender women from Kermanshah, Iran, in 2024. Participants were recruited from obstetric emergency departments, pregnancy clinics, midwifery offices, and gynecologists' practices, presenting with symptoms of incomplete abortion following unsafe procedures.

It is important to note that, while abortion upon request is illegal in Iran and those involved in illegal abortions (e.g., gynecologists, midwives) face legal penalties, women themselves are not punished for attempting abortion. Consequently, women often seek medical care at public or private health centers if complications arise after an abortion—either immediately or within the first week following the procedure. For this study, participants were selected from these health centers. Interviews were conducted by a midwife researcher at the respective centers, scheduled in coordination with the women at a mutually convenient time and place.

The methodology used in the study is qualitative with conventional content analysis, which emphasizes that the study is a description of views and an explanation of experiences based on culture and context [19].

### Participants

Inclusion criteria for the study included women being between 15 and 49 years old, being able to speak and understand Persian, being able to give informed consent, living in the greater Kermanshah region and having previously attempted an unsafe abortion with one symptom of it. Sampling was done by targeted sampling method with maximum diversity. The location of the study was chosen based on the agreement between the researcher and the participants, a place that is more convenient for the participants.

### Data collection

The interviews were conducted between April and August 2024 by the first and second authors, both of whom are female. Prior to the interviews, an interview guide was prepared, inspired by Naeem et al. [19] methodological approach. All participants were provided with a research information sheet detailing the purpose of the study, demographic and obstetric information, as well as an informed consent form for review before the interviews.

The interviews started with the open question, "what were the reasons that made you decide to have an unsafe abortion?". Participants generally could not limit their answers to a single reason and sometimes even provided additional reasons for the question, which would make it difficult to identify the "main" reason. Therefore, the answers to both questions were combined to identify all the reasons given by respondents for requesting an abortion. The interview process is based on people's initial answers with probing questions such as "What do you mean?" or "if you could explain more", is used with questions like "do you mean that"… or "do you mean that…" and "can you explain this more clearly?" which made the phenomenon clearer for researchers and participants, it continued. The duration of the individual interview lasted between 45 and 90 min.

### Data analysis

We used the 8-step method proposed by Graneheim and Lundman to analyze qualitative data [20]. Audio recordings were transcribed by the first author and a professional transcriber after the interviews and then translated from Persian to English. The first author conducted a thematic analysis of the in-depth interview content using MAXQDA version 10 software.

The first author read the transcripts and discussed data saturation before collaboratively refining themes. Thematic analysis allowed for the exploration of evolving themes within the data and facilitated the synthesis of overarching themes, themes, and subthemes. Interview content analysis revealed five overarching themes, 5 themes, and 17 subthemes, as described in Table 1: Thematic content analysis of the interview transcripts (*n* = 46) was conducted using an inductive approach, whereby the textual material informed and guided the authors in identifying and constructing themes aligned with the study's aim. To ensure the validity and reliability of the qualitative data, we applied Guba and Lincoln's four criteria: Credibility, Dependability, Confirmability, and Transparency [21].

**Table 1:** Themes and sub-themes extracted from Iranian women's perceptions regarding the reasons for illegal abortion

Themes	Subthemes	The number of instances each subtheme came up during the interviews (*n* = 46)
Financial hardship and uncertainty about the future	Lack of Basic Necessities and Comforts of Life	14
	Spousal Unemployment and Job Insecurity	11 and 4
	Fears for the Child’s Future	9
Pursuit of a prosperous life	Welfare in childlessness or only child	5
	Individual self-actualization	7
Unstable marital relationship	Wrong Choice of Marriage	12
	Early Married Life	6
	Temporary Marriage	5
Health and fertility issues	Short Interpregnancy Intervals	6
	Unplanned Pregnancy	13
	Negative Childbirth Experience	4
	Fear of Fetal Anomaly	9
	Unhealthy Women or Men	7
Cultural factors	Stigma of Having Children at an Older Age	7
	Illegitimate Pregnancy	9
	Gender-Based Norms (Preference for Sons over Daughters)	6

In this research, member checks and peer checks were used to ensure credibility. Also, prolonged engagement with the phenomenon and continuous investigation (Prolonged engagement) will be other measures that were taken to increase the validity of the findings of this study. To check the dependability, the data obtained from the interviews were reviewed and revised by the third author after implementation and coding. In 95% of cases, the results were similar to the analysis and coding of the research. In the confirmability stage, the researcher tried to write all the steps of the research so that other researchers could follow the data. This proves that there was no bias in the process. Covering a wide range of participants in terms of age, occupation, educational, economic, and social levels provided an effort towards the transferability of the findings to be evaluated and judged by others.

### Ethical considerations

All participants provided written informed consent and were assured of confidentiality and anonymity. Data, audiotapes, and notes were coded numerically to protect identities. Interviews were scheduled at participants’ convenience, and voice recordings were deleted after data analysis and completion of the study [22].

## Results

A total of 46 pregnant women with incomplete abortion signs after unsafe abortion, with ages ranging from 17 to 49 years (mean: 28.62), participated in this study. Most participants were homemaker (31 out of 46). To ensure maximum variation, participants were selected from various age groups, parity and socioeconomic situations to obtain diversity in experiences and perceptions. The quotes represent the participants' expressions about abortion. Participants are identified using fictional names, but their number of pregnancies (G) and age are accurately represented.

Five themes and 16 subthemes were generated from the perceptions of Iranian pregnant women regarding reasons for deciding to have illegal abortions. These themes included economic problems and uncertainty with the future, luxurious life, unstable marital relationship, health and fertility issues, and cultural issues (Table 1).

### Theme 1: Financial hardship and uncertainty about the future

One of the reasons women seek illegal abortions is the overwhelming financial difficulties they face, combined with deep uncertainty about the future. Over two-thirds of the women in this study reported economic-related challenges. This theme highlights three main subthemes*: Lack of Basic Necessities and Comforts of Life, Spousal Unemployment* and *Job Insecurity and Fears for the Child’s Future.* These factors contribute to a sense of helplessness, making the idea of bringing a child into their current situation seem impossible and irresponsible.

#### Lack of basic necessities and comforts of life

Many women described their struggles to meet even the most basic necessities for daily life. For them, having a child was not only an emotional decision but also a practical one, heavily influenced by their financial limitations. The thought of adding the expenses of raising a child on top of their existing financial burdens seemed unmanageable, leading many to the difficult decision to seek an abortion. Financial issues were described by the participants as:

"Although I would like to have a baby, I don't even have the money to buy even baby diapers (with Choke up and crying), Bring a child to be miserable." (Mina, G1, and 21years old)

This testimony reflects the pain of being caught between the personal with to have a child and the harsh reality of not being able to provide for them the basic necessities. The use of phrases like "choked up and crying" emphasizes how deeply this decision affects the women on a personal level, showing that it is not one made lightly, but out of necessity.

Similarly, another participant shared her view and express:

"Due to economic problems, lack of money, and the fact that we cannot afford the basic expenses of our lives, I work alone, but the money is still lacking." (Ziba, G2, and 35 years old)

This highlights a common situation where, despite the woman's best efforts to contribute financially, it is still not enough to sustain the family. The ongoing financial struggle creates a sense of helplessness, reinforcing the belief that bringing a child into such circumstances would only lead to more hardship.

"Because of our economic problems, lack of money, and the fact that my husband is a worker, we cannot afford our own expenses. We also do not have a house or a car, so what will happen if we have another child?" (Elahe, G2, and 25 years old).

This statement not only points to the current financial instability but also shows the anxiety about long-term security. The absence of stable assets like a house or car creates additional stress, making it difficult to imagine raising another child when they are already struggling with their own needs.

#### Spousal unemployment and job insecurity

A significant number of women also mentioned their spouses' unemployment or the fear that their spouse might lose their job as a reason for seeking an abortion. The insecurity of not knowing whether they could continue to rely on their spouse’s income added to their overall financial anxiety. Many women whose partners had unstable employment described how this uncertainty left them feeling unable to plan for the future or take on the responsibility of a child.

"My husband's salary is low and he has an employment contract, with this bad economic situation in the country, he may be terminated at any moment. In the same way, we have financial problems, the child has expenses, how can I cover his living expenses if my husband becomes unemployed?" (Maryam, G1, and 22 years old).

This quote illustrates the fragile nature of financial stability in many families. Even those with current employment worry that they could lose their jobs at any moment, leaving them without any means of support. The fear of future unemployment can be just as paralyzing as current financial difficulties, making it feel irresponsible to bring a child into such an unpredictable situation. For these women, the decision to seek an abortion is not just about present conditions but also about their inability to provide long-term stability for their children. The constant threat of job loss and the lack of financial security in their lives create an environment where having a child seems like an enormous risk.

#### Fears for the child’s future

In addition to the immediate financial struggles, many women expressed concerns about the broader future that their children would face. The economic instability in the country, combined with environmental degradation and political uncertainty, led many women to question whether it was ethical to bring a child into such a world. These concerns go beyond personal financial issues and speak to a broader sense of societal hopelessness.

“Why should I bring a child? Do you see the economic conditions of the country? In a few years, there will be no water, no electricity, nothing else. A wise person does not have children. Whoever brings a child should be responsible for his future. Are you giving birth to a child in this situation of despair?” (Deli, G1, 29 years old)

This reflects a deep sense of despair about the future, not only for themselves but for society as a whole. The participant points to environmental issues like the lack of water and electricity, as well as a general decline in living conditions, as reasons to avoid having children. For many women, the thought of raising a child in such an uncertain and potentially dangerous future feels not only unwise but also unfair to the child. This broader perspective shows that the decision to seek an abortion is not just about individual circumstances but is also influenced by larger societal factors. Many women feel that the future is bleak and that bringing a child into the world under these conditions would be an irresponsible and immoral action.

### Theme 2: Pursuit of a prosperous life

Another significant reason for seeking illegal abortions is the desire to maintain or achieve well-being. For some women, the prospect of having a child or adding another to their family is seen as a threat to their personal well-being and quality of life. This theme reflects two sub-themes: welfare in childlessness or having only one child, and individual self-actualization. These women view abortion not merely as a response to immediate financial stress, but as a means to preserve or enhance their desired standard of living and to focus on their personal growth and aspirations.

In this context, " Prosperous" does not necessarily refer to excessive wealth but rather to a lifestyle that allows comfort, personal freedom, and opportunities for personal development. The narratives of the participants show how their desire for control over their life circumstances and ambitions leads them to make decisions about family planning in ways that prioritize their vision of a good life over the societal expectations of motherhood.

#### Welfare in childlessness or only child

For some women, the presence of another child is viewed as an obstacle to maintaining their lifestyle. They associate having more children with sacrificing personal comfort, financial security, and the ability to enjoy life’s pleasures. In this sense, abortion becomes a way to safeguard their welfare and maintain a balance in their lives that allows for leisure, freedom, and self-care.

"I have a luxurious and good life; I am sick of ruining it with the presence of a child. I had to abort it at any cost." (Sogol, G1, 31 years old).

This quote highlights how some women feel that having children disrupts their pursuit of a comfortable, well-ordered life. The idea of maintaining a lifestyle that they have worked hard to achieve outweighs the desire to expand their family.

Another participant shared a similar view, emphasizing how a child is perceived as a barrier to a peaceful and enjoyable life:

"A child is an obstacle to a peaceful life. My husband also agrees with this. Bringing a child means canceling the fun and pleasures of two people." (Hoda, G1, 28 years old).

In this case, the decision is not just about the woman’s personal welfare but also the couple’s shared lifestyle, with both partners agreeing that raising a child would require them to sacrifice the pleasures and freedom they currently enjoy.

"I don't want to have another child, this one child is enough for me, I want my child to be an only child so that I can better reach him and create a luxury life for him." (Samar, G2, 39 years old).

Here, the idea of luxury is not only about personal well-being but also about being able to provide an enhanced life for their current child. For these women, limiting family size is a deliberate strategy to ensure that the resources and attention they provide to their only child are maximized, allowing them to craft an ideal upbringing.

#### Individual self-actualization

In addition to the desire for material comfort, some women’s decision to have an abortion is driven by their ambition for personal growth and self-fulfillment. These women see motherhood, especially having additional children, as a hindrance to their ability to achieve their professional, academic, or personal goals. For them, abortion represents a way to maintain control over their lives and to continue pursuing their aspirations without the constraints of motherhood.

"I plan to continue my studies at the doctoral level next year, definitely if my pregnancy continues, my academic progress will stop. I have long and distant dreams…". (Henas, G1, 29 years old).

### Theme 3: Unstable marital relationship

In this study many pregnant women expressed that an unstable marital relationship was also one of the reasons behind their decision to seek an abortion. These unstable relationships were linked to several factors, including a wrong choice of spouse, dissatisfaction with married life, early marriage, and temporary or concubine marriages. Each of these factors highlights the complexities of marital instability and how it influences decisions regarding pregnancy and parenthood.

#### Wrong choice of marriage

Several women reported that their marriages were unstable because they believed they had made the wrong choice of spouse. For these women, the realization that their partner was not the right one led them to believe that having a child would only deepen their marital problems and further complicate their lives. They feared that bringing a child into an already unstable relationship would cause unnecessary hardship for both themselves and the child.

One woman shared her experience:

"Our marriage was a mistake from the beginning. He is not my favorite person. I think about divorce every moment, but our society and family do not accept divorce at all. I should not make a child miserable. Even if I don't get a divorce, I will never have a child with this man. That's why I didn't tell my husband about my pregnancy and I bought misoprostol pills from the pharmacy and aborted it..." (Sara, G1, 26 Years old).

This statement reflects not only the regret over her choice of spouse but also the societal pressure to remain in a marriage, even if it is unfulfilling. In this case, the woman felt trapped between her unhappiness in the marriage and her cultural and familial obligations, choosing abortion as a way to avoid further entanglement.

Another key reason for marital instability and the decision to terminate a pregnancy was dissatisfaction with married life. Many women reported feeling neglected by their husbands or living in environments where there was a lack of love, care, and responsibility. In these situations, adding a child to an already challenging situation was seen as worsening the emotional and financial strain on the family.

One participant explained:

"I didn't want to get pregnant because of the problems I had with my husbund. We are all fighting, and we don't have any agreement; he is a careless person. Why should I add a child to this bad situation so that he will be miserable like me?" (Ida, G1, and 31 years old).

This highlights the fear of repeating cycles of misery, where a child could potentially suffer the same emotional neglect that the woman herself was experiencing. The decision to have an abortion was, in many cases, seen as an act of protection for the unborn child.

Another woman expressed a similar sentiment:

"I am not satisfied with my husband; he is a wild person, and he doesn't care about me at all. I have to work because of my children, otherwise, they will die of hunger. I can't have another child in this situation." (Saba, G3, and 41 years old).

In this case, financial insecurity compounded the emotional neglect in the marriage. The burden of providing for existing children without the support of her husband made the idea of having another child unbearable.

#### Early married life

Some women felt that the early years of marriage were too unstable to bring a child into the world. These women were uncertain about the future of their marriages and feared that having a child too soon might complicate their lives if the marriage ended in divorce. For these women, the instability of early married life made them cautious about pregnancy, and in some cases, they opted for abortion if they became pregnant.

One participant shared her decision-making process by expressing:

"My husband doesn't know about my pregnancy. We just got married, and we always argue. I don't plan to get pregnant until a few years have passed since our marriage. I'll do it after life is stable." (Sanaz, G1, and 19 years old).

This statement illustrates the hesitation some women experience in the early stages of marriage, when uncertainties about the future and occasional disagreements are present. For these women, delaying parenthood until achieving stability was considered important for creating a better foundation for their family.

#### Temporary marriage

Temporary or concubine marriage, a practice common among some Muslim communities, was another factor contributing to decisions about abortion. In this form of marriage, a couple is married for a fixed period, and once that period ends, the marriage is automatically dissolved. Since these marriages are often not recognized legally or socially, many couples choose not to have children. If a pregnancy does occur, abortion is frequently seen as the only option, as the future of the relationship and the child remains uncertain.

One participant explained her situation as:

"Because our marriage was not official and I was a concubine and I did not want anyone to find out about this issue, and my husband had temporarily made me a concubine and had no intention of officially marrying, I did not want to keep the child, and grow up him without a father." (Elham. G1, and 37 years old).

For this woman, the stigma of a temporary marriage and the uncertainty of her husband's commitment influenced her decision to abort the pregnancy. Raising a child without the security of an official, long-term marriage seemed both socially and emotionally challenging.

Another participant explained briefly by saying:

"You cannot have children with a temporary marriage." (Hana, G1, and 28 years old).

This reflects a common understanding among women in temporary marriages—that the nature of these unions is incompatible with the responsibilities of parenthood. In such cases, abortion becomes a practical solution to avoid complications that may arise from an impermanent relationship.

### Theme 4: Health and fertility issues

Health and fertility concerns were significant factors driving women to seek abortions. These concerns included both physical and mental health issues, as well as circumstances where the timing of the pregnancy or the condition of the fetus led women to feel that continuing with the pregnancy would be unwise. This theme is divided into five subcategories: short interpregnancy intervals, unplanned pregnancy, negative childbirth experience, fear of fetal anomaly, and health problems affecting either the woman or her partner.

### Short interpregnancy intervals

One of the key reasons women cited for seeking an abortion was the short time between pregnancies. Many women felt that their bodies had not yet recovered from their previous pregnancies, and the idea of going through another so soon was overwhelming. They also expressed concern about their ability to properly care for their existing children while managing the demands of a new baby.

"Because I have two young children, my little one is one-year-old, and I can't afford to have another new child and take care of him, so I aborted him/her. And that my body is not yet ready for a new pregnancy." (Elnaz, G3, and 30 years old).

This statement reflects both the physical and emotional toll of closely spaced pregnancies. The participant’s decision was driven by the exhaustion of caring for two small children and the recognition that her body needed time to heal before she could consider having another child.

Similarly, another participant expressed:

"I don't want any more children; I have a breastfeeding child that is difficult to have another child." (Mahshid, G2, and 31 years old).

Here, the practical challenges of raising a breastfeeding infant made the thought of another pregnancy seem untenable. For many women, the demands of caring for a young child already take up their time and energy, making it impossible to imagine caring for a newborn so soon after.

#### Unplanned pregnancy

Some women explained that although they had planned to have more children in the future, they were not ready to be pregnant at that moment. Their decision to abort was motivated by a desire to be in a better physical, emotional, or financial state before having another child.

"I plan to have another child, but not now. I was not ready for pregnancy now, mentally I wanted to feel better and when I have a baby, to be satisfied with everything, to feel good, to be in better condition." (Arezoo, G2, 27 years old).

This highlights the importance of mental readiness and well-being in the decision to carry a pregnancy to term. For these women, timing is crucial, and they want to ensure that they are in the best possible position to provide a stable, loving environment for their future child.

#### Negative childbirth experience

Another common reason for seeking an abortion was a negative experience with a previous childbirth. The physical pain, fear, and trauma associated with past births left many women feeling unwilling to go through the process again.

"The thought of giving birth is a nightmare for me. I am not ready to experience that pain again. I told my husband that I will never give birth again. This made me look for an abortion and buy pills." (Kowsar, G3, and 39 years old).

For this woman, the psychological impact of her previous childbirth was so profound that it shaped her decision to avoid future pregnancies altogether. Another women expressed:

"I was very scared and bothered during my first birth and I aborted the baby because of the extreme fear of giving birth." (Zahra, G2, and 30 years).

This demonstrates how past trauma can influence a woman’s decision to seek an abortion. The fear of reliving the intense pain and anxiety associated with childbirth can be overwhelming, leading some women to view abortion as the only way to avoid that experience again.

#### Fear of fetal anomaly

Another significant factor leading to unsafe abortions was the fear of fetal anomalies or abnormalities. With changes in policies surrounding fetal health screening in Iran, many women feared that they were not receiving accurate information about the health of their unborn child. This uncertainty, combined with the potential stigma associated with raising a child with a disability, led some women to terminate their pregnancies.

"I did an ultrasound and he said that my baby had problems with his face and legs. The forensic doctor did not permit abortion and said that the problem was not big. I didn't want to keep the child; it was tough for me to have everyone say that the child is retarded." (Maejan, G2, and 46 years old).

This quote reflects the emotional distress associated with the possibility of raising a child with a disability. The fear of societal judgment and the perceived difficulty of caring for a child with special needs contributed to the woman’s decision to seek an abortion, even when medical professionals downplayed the severity of the anomaly.

#### Unhealthy women or men

Health issues affecting either the woman or her partner were also cited as reasons for seeking an abortion. Physical or mental illness, as well as addiction, made many women feel that they were not in a position to care for a child, either because they could not manage the demands of parenthood or because their partners were not capable of providing support.

"Both my husband and I take neuroleptics and we were not mentally capable of protecting a small child, we wanted to have an abortion. We went to a private midwife's office and she gave me pills and I aborted." (Robab, G3, and 49 years old).

This case highlights how mental health challenges can deeply affect a couple’s decision-making process around pregnancy. For this couple, their mental health issues made them feel unfit to raise a child, leading them to pursue an abortion as a responsible choice.

Another woman expressed her experience with an addicted husband:

"My husband is an addict; why should I not abort the child? My life is like this." (Shabnam, G2, and 37 years old).

For women in situations where addiction is present, the decision to have an abortion may be seen as a way to protect the child from growing up in a harmful environment. The instability caused by addiction makes the prospect of raising a child seem not only challenging but also unfair to the child.

### Theme 5: Cultural factors

Cultural norms and societal expectations play a significant role in shaping fertility behaviors and decisions regarding abortion. In some regions of Iran, cultural attitudes towards mistimed pregnancies, pregnancies from illicit relationships, and even the gender of the fetus exert immense pressure on women. These pressures often compel women to seek abortions in order to avoid social stigmatization and maintain cultural acceptance. This theme is divided into three subthemes: Stigma of having children at an older age, Illegitimate pregnancy, and Gender-based norms.

#### Stigma of having children at an older age

In many societies, including parts of Iran, there is a stigma attached to women who become pregnant at an older age, often considered to be beyond 45, which is seen as the end of reproductive age. These women face social pressures that label their pregnancies as inappropriate or even shameful. Such societal expectations can heavily influence their decisions to terminate the pregnancy, especially when they feel that their age does not align with cultural ideals of motherhood.

"I am old and I know that it is very difficult to get pregnant at an old age, that's why I didn't want to have a child, and I knew that there were rumors behind me that at this age, having a child is not a good idea. My husband also wanted us to abort the baby, he was embarrassed to have a baby at this age, so we went to a local midwife and she performed suction for me." (Sahar, G3, and 49 years old).

This testimony reflects how societal judgment, rumors, and the perception of age-appropriateness for motherhood shape decisions about abortion. For this woman, the pressure was twofold: not only was she concerned about the physical challenges of pregnancy at an older age, but she also felt ashamed due to the stigma surrounding late-life pregnancies. In such cases, the fear of being ridiculed or judged by their community pushes women to opt for abortion as a way to avoid being seen as socially deviant. Cultural ideals of when it is "acceptable" to have children create a narrow window of opportunity for motherhood, outside of which pregnancy is perceived negatively.

#### Illegitimate pregnancy

Pregnancy outside of marriage or within illicit relationships carries severe cultural consequences in many parts of Iran. Women who find themselves in these situations face harsh judgment, ostracism, and potential legal penalties. The fear of societal disgrace, especially in cases where honor and family reputation are at stake, often forces women to seek abortion as the only way to preserve their standing in society.

"The current pregnancy is from my husband's uncle, with whom we had an illicit relationship, my husband died, that's why we wanted to abort him, and the rest of the family didn't know, because of the honor and prestige issues, it was definitely necessary to have an abortion." (Mitra, G3, and 38 years old).

This story underscores the weight of cultural and familial honor in such decisions. The concept of honor, particularly within extended family dynamics, can be an overwhelming force, leaving women with no other option but to terminate the pregnancy. The risk of public scandal or familial disgrace becomes a driving factor behind the decision, even when the pregnancy might otherwise be desired.

In these cases, abortion is not solely a personal choice but rather a response to the intense pressure to conform to cultural norms surrounding marriage, sexuality, and reputation. The fear of being labeled immoral or bringing shame to the family makes abortion appear as the only viable solution.

#### Gender-Based norms (preference for sons over daughters)

In some regions, the cultural preference for male children persists, and this gender-based norm often influences decisions about abortion. Families may opt for abortion if they find out the fetus is female, especially if they already have daughters. This cultural preference for sons is deeply rooted in traditional values and often reinforced by the desire to carry on the family name or to meet societal expectations of masculinity.

"I have two daughters and I wanted the third one to be a boy. I had an abortion because I wanted to do IVF to determine the sex, but I was afraid that it would be a girl again. My husband did not like the child to be a girl, he also agreed that we should abort the child and that we should do IVF to have a boy. That's why he went to buy pills smuggled across the border." (Zohreh, G3, and 29 years old).

This reveals how cultural expectations about gender can overshadow the intrinsic value of a child. The participant’s decision to abort was driven by a fear of disappointment if the child was another girl, as well as by societal and familial pressures to produce a male heir. In this case, the husband’s involvement in procuring abortion pills emphasizes the joint decision to prioritize cultural ideals of masculinity over the pregnancy itself.

Another participant described her dilemma of having a girl:

"My child was a girl, while I had a girl and wanted to have a boy, my husband doesn't like girls either." (Ashraf, G2, and 31 years old).

This highlights the persistence of gender discrimination, where female fetuses are viewed as less desirable simply because of their sex. In such environments, abortion becomes a tool to ensure that only male children are born, perpetuating harmful gender biases and inequalities.

## Discussion

This study highlights the multifaceted factors that influence women’s decisions to pursue illegal abortions. The five themes identified—economic hardship, unstable marital relationships, the desire for a prosperous life, health and fertility issues, and cultural pressures—offer insight into how broader societal forces, personal autonomy, and ethical considerations intersect. To fully understand these motivations, it is essential to examine how women's reproductive choices are shaped by their social, economic, and cultural environments, often limiting their autonomy [23] and placing them in morally and socially precarious positions.

A particularly significant finding is that over two-thirds of the women cited economic challenges as their primary motivation for seeking an abortion. Iran’s recent economic inflation, sanctions, and restrictive population policies have worsened financial conditions for many families, leading to increased poverty and insecurity [24]. Women in these circumstances may find themselves grappling with difficult decisions regarding pregnancy due to their inability to meet basic needs, aligning with Maslow’s hierarchy of needs [25], which suggests that when basic physiological needs are unmet, individuals' behaviors and decisions are significantly affected. Economic deprivation in this context can be seen as a form of coercion, limiting women's ability to make truly autonomous reproductive decisions [26, 27]. This finding contrasts with studies conducted in wealthier nations, such as Sweden, where the primary cause of abortion tends to be poor relationship dynamics, contraceptive use, unintended pregnancies [28], rather than economic hardship, likely reflecting the differences in social welfare systems between the countries [29].

Beyond economic factors, unstable marital relationships emerged as a strong reason for abortion in this study, especially in cases where women experienced abusive, neglectful, or unsupportive partners. These findings align with other studies, such as Chae's research across 14 countries, which identified poor relationships and partner characteristics as common reasons for seeking abortion [29–31]. In some cases, abortion is viewed as an act of self-preservation, particularly in abusive relationships where women may feel trapped by societal expectations that discourage divorce or single motherhood. Feminist ethics emphasizes that decisions about abortion in such contexts must be understood in relation to broader relational and societal pressures, where autonomy is deeply embedded in power dynamics within relationships [29, 30].

Additionally, the desire for personal development and a prosperous life further complicates the ethical discussion. Some women in the study expressed that having a child would conflict with their aspirations for self-actualization, which resonates with existentialist philosophy. Existentialism emphasizes the importance of individual freedom, autonomy, and the pursuit of personal fulfillment, suggesting that individuals are responsible for creating meaning in their lives [32, 33]. For these women, the decision to delay or forgo childbearing was framed as an assertion of their autonomy and their right to prioritize their own goals in life, whether personal, professional, or relational. Furthermore, existentialist thought often challenges societal norms and pressures, aligning with the experiences of women in our study who felt constrained by cultural expectations surrounding motherhood [34, 35]. This perspective underscores the tension between societal prescriptions of women's roles and their individual desires for freedom and self-definition.

Pronatalism is a complex ideology that emphasizes the importance of childbirth and motherhood as central to societal values and individual identity. This discourse often manifests in various cultural, religious, and national contexts, influencing policies and personal choices regarding family size and reproductive health. The implications of pronatalist ideology are particularly pronounced for women, who frequently find their social worth tied to their ability to bear children. Such ideological frameworks can exacerbate the already significant cultural pressures identified in this study, framing motherhood not only as a societal expectation but as a measure of a woman’s identity and value. This can lead to internalized guilt and societal judgment when women deviate from these norms, particularly in cases of abortion, thereby further constraining reproductive choices and autonomy.

In addition, feminist ethics complements this framework by highlighting the relational and situational factors influencing moral decisions [36, 37]. From this perspective, the women's considerations around childbearing reflect an ethical negotiation that values both their aspirations and their potential responsibilities, challenging the traditional view of motherhood as a universal moral obligation. However, this raises the moral question of responsibility, particularly when weighing individual goals against the potential life of a child. While autonomy and personal growth are legitimate reasons for seeking abortion, this tension reflects deeper philosophical questions about freedom, responsibility, and ethical obligations toward future generations [38].

Health and fertility concern also played a central role in motivating abortion decisions, with factors such as short interpregnancy intervals, unplanned pregnancies, negative childbirth experiences, and fear of fetal anomalies contributing to these decisions. The restriction of prenatal screenings and access to family planning services due to Iran's new population policies further exacerbates the situation. By limiting women's access to important medical information, these policies violate their right to informed consent, leading many to seek unsafe abortions. The principle of non-maleficence, which requires medical professionals to prevent harm, becomes particularly relevant in this context, as these policies indirectly increase the risks associated with pregnancy and abortion [8, 39].

Cultural pressures, including the stigma of having children at an older age, illegitimate pregnancies, and gender-based norms, also play a significant role in shaping reproductive decisions. The preference for male children, in particular, continues to drive gender-based abortions, which highlights deep-seated sexism and patriarchal values in certain cultural contexts [40]. This raises an ethical dilemma: while individuals may have the right to make personal decisions regarding reproduction, the practice of aborting based on gender preference perpetuates harmful social norms that devalue women and girls. In the case of illegitimate pregnancies, Iranian law criminalizes sexual relations outside of marriage, creating a paradox where women are punished both for engaging in these relationships and for seeking abortions to avoid social stigma. This sociocultural framework not only denies women reproductive freedom but also subjects them to harsh legal and social consequences, pointing to a broader ethical issue of justice [41].

The results of this study support existing research in Iran that identifies economic problems, health concerns, and cultural norms as major drivers of unsafe abortion [24, 42]. The ongoing economic crisis, coupled with restrictive reproductive policies, has forced many women into unsafe and illegal abortions [24]. Critics argue that the limited access to contraceptives and prenatal screenings, combined with the societal stigma surrounding illegitimate pregnancies and gender preferences, has created a hostile environment for reproductive decision-making. Addressing these challenges requires policymakers and healthcare providers to consider the ethical, social, and legal frameworks that constrain women’s reproductive autonomy. Upholding ethical principles such as autonomy, justice, and informed consent is crucial in creating a more just and supportive environment for women facing these difficult decisions [29].

### Strengthen

This qualitative study, conducted after the implementation of the family and youth laws in Iran, uniquely captures the experiences of women who have undergone abortions. The study offers valuable insights for policymakers, planners, and sociologists working in the field of population and reproductive health in Iran, serving as a critical resource for addressing the challenges faced by women in restrictive environments.

### Limitation

This study was conducted with interviews with women who had an illegal abortion but went to the hospital with abortion symptoms due to an incomplete abortion. As such, it does not provide any information about those who have had illegal abortions and had no problems afterwards. The results presented in this paper only reflect the perceptions of the women who had an induced abortion, not those of their partners. The paper is based on qualitative data that provides insights into factors influencing abortion decision-making. Since the sample included in the study is not representative of the population of reproductive-age women in Iran, the results cannot be generalized.

## Conclusion

In conclusion, this study highlights that women’s decisions to seek illegal abortions are influenced by a complex interplay of economic, social, health, and cultural factors. The key motivations include economic struggles, uncertainty about the future, the desire for a more affluent lifestyle, unstable marital relationships, health and fertility challenges, and societal pressures. Among these, economic hardship and health-related concerns emerged as the most significant drivers of unsafe abortions in Iran, reflecting the country's worsening economic conditions and restrictive population policies that limit access to reproductive health services. These factors, combined with relational dynamics and ingrained societal norms, heavily shape women’s reproductive decisions, often compromising their autonomy and placing them in difficult moral and social situations.

To address these issues, it is essential for policymakers and healthcare providers to consider the broader social, cultural, and legal frameworks that restrict women’s choices. Upholding ethical principles such as autonomy, justice, and informed consent is crucial in creating a more supportive environment for reproductive decision-making.

## Data Availability

No datasets were generated or analysed during the current study.
